# Marked Genotype Diversity among Reoviruses Isolated from Chicken in Selected East-Central European Countries

**DOI:** 10.3390/ani13132137

**Published:** 2023-06-28

**Authors:** Bence Gál, Renáta Varga-Kugler, Katalin Ihász, Eszter Kaszab, Marianna Domán, Szilvia Farkas, Krisztián Bányai

**Affiliations:** 1 Intervet Hungária Kft, Lechner Ödön fasor 10/b, H-1095 Budapest, Hungary; bence.gal@merck.com; 2 Veterinary Medical Research Institute, Hungária krt. 21, H-1143 Budapest, Hungary; kugler.renata@gmail.com (R.V.-K.); ihaszkatalinn@gmail.com (K.I.); eszter.kaszab@gmail.com (E.K.); doman.marianna@vmri.hu (M.D.); 3 National Laboratory for Infectious Animal Diseases, Antimicrobial Resistance, Veterinary Public Health and Food Chain Safety, Hungária krt. 21, H-1143, Budapest, Hungary; 4 Department of Obstetrics and Food Animal Medicine Clinic, University of Veterinary Medicine, István utca 2, H-1078 Budapest, Hungary; fszilvi@yahoo.com; 5 Department of Pharmacology and Toxicology, University of Veterinary Medicine, István utca 2, H-1078 Budapest, Hungary

**Keywords:** *Avian orthoreovirus*, phylogenetic analysis, genotyping

## Abstract

**Simple Summary:**

*Avian orthoreoviruses* cause significant losses to the poultry industry worldwide. Reovirus-associated diseases in chickens are frequently reported even in vaccinated flocks due to the emergence and spread of novel strains. Herein we studied the genetic features of reovirus strains originating from parts of East-Central Europe. The isolated strains from the study area displayed large genetic variability and shared low genetic similarity with vaccine strains. These findings highlight the importance of immune pressure from vaccination in the epizootiology of reovirus infection in chickens.

**Abstract:**

The concern that the vaccines currently used against *Avian orthoreovirus* (ARV) infections are less efficient in the field justifies the need for the close monitoring of circulating ARV strains. In this study, we collected necropsy samples from various chicken breeds and tested for ARV by virus isolation, RT-PCR assay and sequence analysis. ARVs were isolated from birds showing runting-stunting syndrome, uneven growth, lameness or increased mortality, with relative detection rates of 38%, 35%, 6% and 25%, respectively. Partial σC gene sequences were determined for nearly 90% of ARV isolates. The isolates could be classified into one of the major genetic clusters. Interestingly, cluster 2 and cluster 5 were isolated from vaccinated broiler breeders, while clusters 1 to 4 were isolated from unvaccinated broilers. The isolates shared less than 75% amino acid identities with the vaccine strains (range, 44.3–74.6%). This study reaffirms the global distribution of the major genetic clusters of ARVs in chicken. The diversity of ARV strains isolated from unvaccinated broilers was greater than those detected from vaccinated animals, however, the relative importance of passive and active immunity on the selection of novel strains in different chicken breeds needs to be better understood.

## 1. Introduction

*Avian orthoreoviruses* (ARVs) are ubiquitous in domestic and wild birds [1]. ARV infections cause significant losses to the poultry industry worldwide, infecting chickens, turkeys and waterfowl. The observation that ARV infection may occur repeatedly despite routine vaccination of chicken flocks and many of the emerging strains share low similarity with vaccine strains renewed research interest in ARV [2,3,4,5,6,7,8,9]. 

ARVs (family *Spinareoviridae*, genus *Orthoreovirus*) are characterized by medium-sized, non-enveloped icosahedral virion enclosing the double-stranded RNA genome. The ten genomic RNA segments encode eight structural and three or four non-structural proteins. The structural protein σC, is responsible for the attachment to the host cell and virus neutralization [10]. The σC protein is characterized by marked genetic and antigenic variability and has a key role in the development of the host immune response, therefore it is the primary target of vaccine development. 

Although ARV has been detected in association with numerous disease conditions such as viral arthritis/tenosynovitis, runting-stunting syndrome, hepatitis, myocarditis, enteric and respiratory diseases and central nervous system infections, the virus can be isolated from healthy birds as well [1,11,12]. The most common forms of ARV infections in broilers are runting-stunting syndrome and viral arthritis/tenosynovitis. Runting-stunting syndrome is common in 2–3 weeks old chickens and is characterized by uneven growth rate, slower development, bone formation disorder and abnormal feathering. Numerous viruses have been associated with the disease (such as reovirus, astrovirus, coronavirus, rotavirus, and adenovirus); hence, the role of ARV in the complex disease etiology is not well understood [1]. The only disease for which the pathogenic role of ARVs has been proven is viral arthritis/tenosynovitis, which mainly affects 4–16-weeks old broilers and breeders. The disease is characterized by inflammation of the tibiotarsal-tarsometatarsal joints and the rupture of the gastrocnemius tendon resulting in lameness [13]. Although tenosynovitis can be efficiently controlled by vaccination using live attenuated or inactivated whole virus vaccines, from the 2010s onward an increasing number of tenosynovitis cases were reported even from vaccinated flocks [2,3,6,9]. These cases were often associated with newly emerging variants that showed high genetic diversity and differed antigenically when compared to vaccine strains. The genetic diversity may affect the antigenic structure enabling these new variants to escape vaccine-induced immunity. 

A more precise understanding of the distribution of emerging new variants as well as the driving forces of genetic diversification among chicken-origin ARVs requires a characterization of the sequence and genetic relationship of a large number of strains. The aim of our study was to gather missing information on the genetic diversity of ARVs currently circulating in the East-Central European region with a focus on Hungary. For this purpose, we collected organ samples, isolated ARV strains and then characterized the isolates by sequencing and phylogenetic analysis of the genomic region encoding σC protein. 

## 2. Materials and Methods

### 2.1. Samples, Data, and Laboratory Processing

Samples were collected from domestic poultry flocks in East-Central European countries (Hungary, Romania, Russia and Ukraine) in 2016. As part of this passive surveillance, information on the general features (such as breed types, age, health status, geographic origin, sample type, vaccination history), on the clinical history (morbidity, mortality) and on the gross pathology were collected by local field veterinarians (see Supplementary File). The nature of sample and data sharing implies that the dataset was occasionally incomplete. Samples were collected only from succumbed animals. Laboratory methods included virus isolation on permissive cell line and then, upon development of the typical cytopathogenic effect (i.e., syncytium formation), confirmation of reovirus infection by virus-specific RT-PCR. Pathogens other than reovirus were not sought in the sample collection.

### 2.2. Virus Isolation

Each organ sample was homogenized individually in sterile phosphate buffered saline using Qiagen TissueLyser II at 50 Hz for 3–5 min. After centrifugation at 10,000× *g* (for 5 min at 4 °C) supernatants were filtered through a 0.45 µm PES syringe filter. For virus isolation, filtered homogenates were inoculated in a four-member ten-fold dilution on chicken hepatocellular carcinoma cell line LMH (Leghorn male hepatoma, ATCC CRL-2117). LMH cells were cultured in DMEM medium (Lonza) supplemented with 5% fetal bovine serum (Gibco) and 1% antibiotic/antimycotic solution (Lonza) in a 37 °C incubator with constant supply of 5% CO_2_. Upon giant cell formation, the cytopathogenic effect (CPE) characteristic of ARV, could be observed (typically after 5–6 days), the isolates were propagated in a 25 cm^2^ cell culture flask. When CPE was not seen following the incubation period, blind passages were carried out; after three blind passages, CPE-negative samples were discarded. 

### 2.3. Nucleic Acid Extraction and Amplification of σC Gene

After three freezing/thawing cycles, cell culture suspensions were centrifuged at 10,000× *g* for 5 min at 4 °C. Viral RNA was extracted from the supernatants using Direct-zol RNA Miniprep (Zymo Research) according to the manufacturer’s recommendations. Chicken reovirus σC gene-specific PCR assay was performed applying QIAGEN One-Step RT-PCR Kit. The primer pair S1_all_F3 (Fw) 5′-GATACTSTCNTTGACTTCGA-3′ and S1_all_R2 (Rev) 5′-TCGATGCCSGTACGCACGGT-3′ were designed to amplify a genomic region of ~900 nucleotide (nt) in length (position, nt 674 to 1603) on the S1 segment of S1133 chicken reovirus vaccine seed strain (accession number KF741762) including the partial sequence of the σC gene. Sanger sequencing of PCR products was carried out on an automated sequence analyzer (ABI PRISM^®^ 3100-Avant Genetic Analyzer) using BigDye Terminator v3.1 Cycle Sequencing Kit (Thermo Fisher Scientific). The newly generated sequences were deposited in GenBank (OP816667–OP816738).

### 2.4. Data Analysis

Sanger sequencing reads were assembled and edited using BioEdit and AliView [14,15]. BLASTn and BLASTx algorithms were used to identify homologous genes among sequences deposited in GenBank [16]. Codon-based multiple sequence alignment was generated using the Muscle algorithm within the Geneious Prime software 2023.1.1 (Biomatters Ltd.) [17]. Phylogenetic analysis was performed and sequence identity values were calculated using the MEGAX package [18]. The gene-specific substitution model was evaluated, and the best-fit model was selected based on the Bayesian information criterion. The maximum-likelihood tree was generated, and tree topology was validated by bootstrap analysis (100) as implemented in MEGAX. Pairwise distance matrix was generated using the SDTv1.2 software [19]. 

## 3. Results

### 3.1. Incidence of ARVs in Chicken

**Figure 1:**
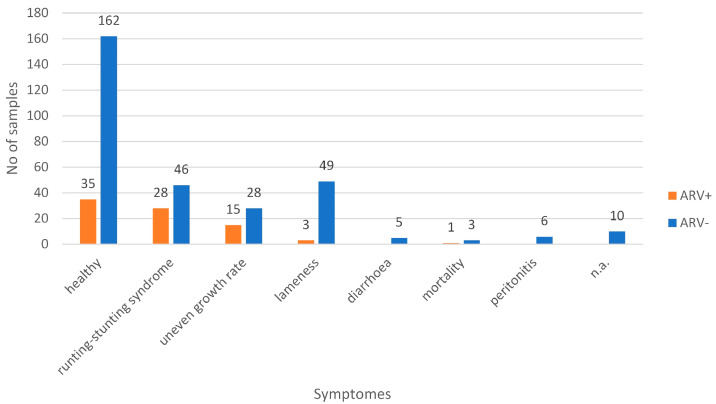
Ratio of ARV-positive (RT-PCR confirmed) and -negative samples according to symptoms.

A total of 391 samples originated from 38 chicken farms, located in Hungary (*n* = 346; 31 farms), Romania (*n* = 36; four farms), Ukraine (*n* = 6; two farms) and Russia (*n* = 3; one farm) were collected in a one-year period. Broilers (*n* = 248), broiler breeders (*n* = 137) and layers (*n* = 6) were sampled. Both healthy animals and flocks with clinical symptoms observed in association with ARV infection were included in the survey. Approximately, half of the samples (*n* = 197) were collected from otherwise healthy flocks, while the remainders were collected from flocks with excess mortality (*n* = 4) and various health issues, including peritonitis (*n* = 6), diarrhea (*n* = 5), runting-stunting syndrome (*n* = 74), lameness (*n* = 52), and uneven growth rate without any other clinical sign (*n* = 43). No data on health status were available for ten samples (Figure 1).

Small intestine including the caecal tonsils (*n* = 318), tendon with synovial fluid (*n* = 57), proventriculus (*n* = 6), yolk sac (*n* = 5), kidney (*n* = 2), trachea (*n* = 2) and liver (*n* = 1) were collected and used for virus isolation on the monolayer of LMH cells (Table 1). Giant cell formation, the CPE specific to ARV infections, was recorded for 106 samples, whereas most samples (*n* = 285) did not produce CPE on the cell monolayer. As for the country-specific distribution, virus isolation was successful from Hungary, Romania, Ukraine and Russia, respectively, from 87 of 346, 17 of 36, 1 of 6 and 1 of 3 specimens. CPE-positive samples were detected exclusively from the intestine (*n* = 103) and the proventriculus (*n* = 3) (Table 1). No CPE was seen when tendon with synovial fluid, yolk sac, kidney, trachea and liver samples were inoculated onto monolayer of LMH cells. An RT-PCR assay targeting the σC protein coding gene was utilized to identify ARV genomic RNA from nucleic acid extracts of virus isolates showing giant cell formation in a subset of isolates (82 of 106 CPE-positives, 77.4%; a proportion of CPE-positive samples were not further tested by RT-PCR, especially, if these isolates were identified from flocks in which there were already RT-PCR confirmed cases). 

The number of confirmed ARV-positive samples was high in flocks showing clinical signs typical to ARV infection (47/184 samples, 26%). The rate of positive samples was also fairly high in samples obtained from healthy flocks (35/197 samples, 18%), but the difference between the two groups was significant (chi-square value, 16.9; *p* = 0.00004) (Table 2 and Figure 1). ARVs were isolated from birds showing runting-stunting syndrome, uneven growth, lameness or increased mortality, with relative rates of ARV-positive samples being 38%, 35%, 6% and 25%, respectively (Figure 1).

The age of chicken whose samples were collected ranged from 1 day to 51 weeks. Nearly three-quarters of birds (277/391, 71%) were six weeks old or younger; from birds in this age range, a total of 68 ARVs (83%) were isolated. On the other hand, ARVs were not isolated from birds younger than 10 days of age. Of note is that the average age of ARV-positive broiler breeder flocks showed some differences when comparing healthy and diseased flocks of broilers (Table 3). Two broiler breeder flocks from the same farm showing no clinical signs were sampled simultaneously. ARVs were isolated from organ samples of birds at the age of four to six weeks, while birds at age of five to twelve days were negative. Furthermore, the samples of breeder flocks and young progeny broilers were negative for ARV, but at the age of four weeks ARVs could be isolated from the same flock. These findings were consistent with horizontal transmission of ARVs among birds. 

### 3.2. Study Strain Classification, Pathology, Molecular Epidemiology

**Figure 2:**
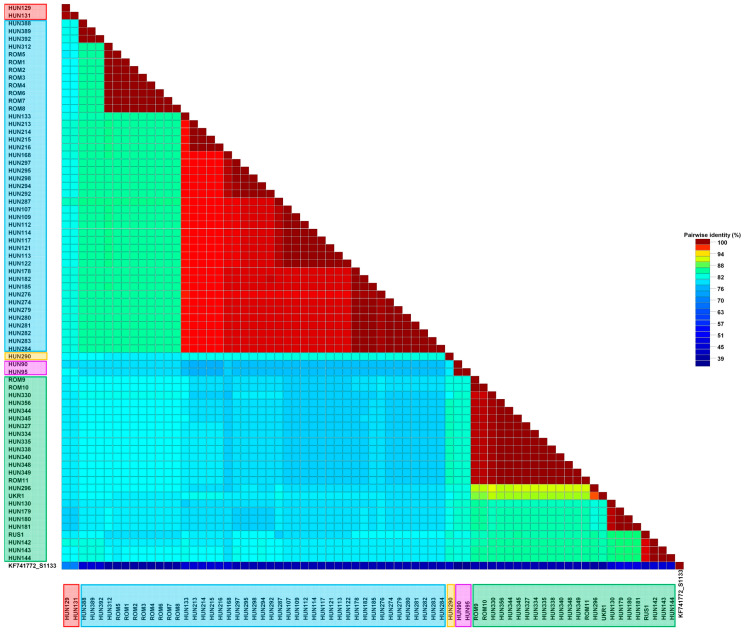
Pairwise identities of the study strains based on a 768 nucleotide long partial sequence of the ORF coding the σC protein. Sequence names are colored by the genetic cluster: cluster 1—red; cluster 2—blue; cluster 3—yellow; cluster 4—green; cluster 5—pink.

An alignment of 768 nt in length was generated from the partial gene encoding the σC protein. Partial sequences were determined for 72 of 82 (87.8%) CPE-positive RT-PCR confirmed isolates. After the closest relatives of study isolates were identified in GenBank through the BLAST engine, pairwise identity values were calculated, and phylogenetic analysis was performed to reveal their genetic relationship. Study isolates showed only moderate nt and amino acid (aa) sequence identities (nt, 49.7–74.5%; aa, 44.3–74.6%) when compared with the vaccine strains S1133, 1733 and 2408, each belonging to cluster 1 ARVs (Figure 2).

**Figure 3:**
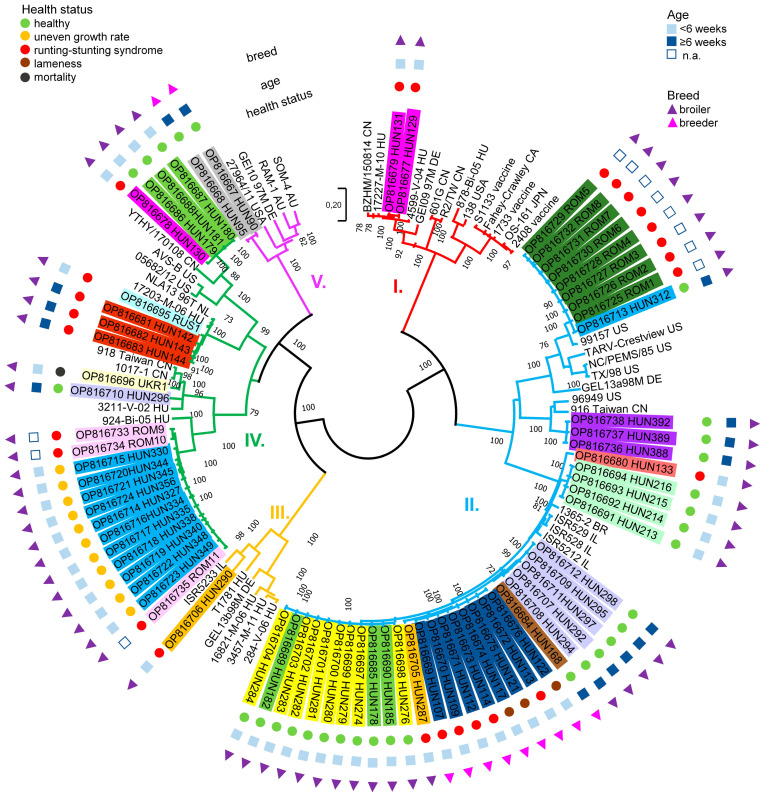
Unrooted phylogenetic tree based on the 768 nucleotide long partial sequence of the ORF coding the σC protein. Isolates originating from the same farm are marked with the same color. Genotypes are marked by the coloring of tree branches (based on Kant et al., 2003 [20]). Phylogenetic tree was calculated by the maximum-likelihood method, bootstrap values greater than 70 are shown at the branch nodes.

**Figure 4:**
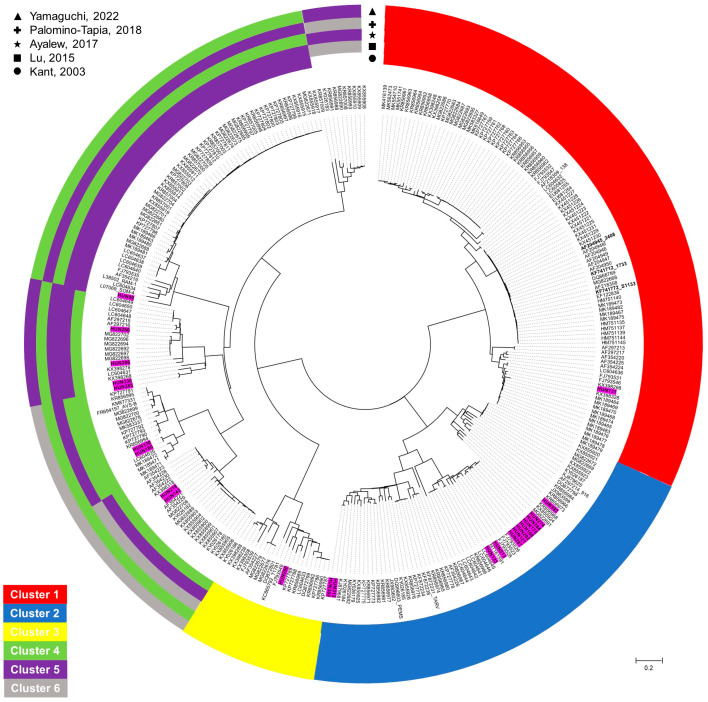
Unrooted phylogenetic tree based on the 768 bp long partial nucleotide sequence of the sigmaC gene of different chicken and turkey origin orthoreoviruses. Strains analyzed in the recent study are marked with pink color. Clusters according to different classification systems are indicated by colored circles. Phylogenetic tree was calculated by maximum-likelihood method. The scale bar is proportional to the genetic distance [2,6,20,21,22].

Based on the sequence identity matrices and phylogenetic analyses of the σC protein coding genomic region, the 72 study strains formed five clusters (cluster 1 to cluster 5). Seeing the lack of consistency in classification, we chose to use the original scheme established by Kant and coworkers [20] (Figure 3 and Figure 4). Study strains within clusters shared at least 60.7% nt and 60.1% aa identity (cluster 1, nt 71.7–99.8%, aa 71.5–100%; cluster 2, nt 64.1–100%, aa 65.8–100%; cluster 3, nt 66.7–97.8%, aa 69.3–97.4%; cluster 4, nt 60.7–100%, aa 60.1–100%; cluster 5, nt 75.2–99.9%, aa 77.6–99.6%) with each other and with the reference strains.

When analyzing the genetic information of ARV strains in relation to clinical presentation of infection, we could not find any association, although the number of samples was relatively low (41 genotyped ARV strains from 15 flocks on 10 farms with ARV associated syndromes, and 31 genotyped strains from 10 healthy flocks of eight farms). Nonetheless, two cluster 1 isolates from the same farm were identified in association with runting-stunting syndrome. Cluster 2 isolates were seen in nine healthy flocks in seven farms (*n* = 25) and were in association with runting-stunting syndrome and lameness in another four farms (*n* = 18). Only a single strain belonging to cluster 3 was identified from a flock with runting-stunting syndrome. Cluster 4 strains were isolated from two healthy flocks (*n* = 4) and from 11 flocks of six farms (*n* = 20) with runting-stunting syndrome, uneven growth and increased mortality. Two cluster 5 strains were isolated from a healthy farm in Hungary (Figure 3).

Cluster 1, 3 and 5 ARVs were detected only in Hungary, cluster 2 ARVs were found in two countries (Hungary and Romania), while cluster 4 ARVs were identified in samples from all four countries. In five cases, we could detect virus strains belonging to different clusters in samples collected at the same time on the same farm (Figure 3). Strains originating from different countries from which samples were collected showed high similarity in some cases. In cluster 2, eight Romanian strains (ROM-1 to ROM-8) and one Hungarian strain (HUN-312) showed 99.59–99.73% nt and 99.56% aa identity. In cluster 4, 11 Hungarian strains (HUN-327, 330, 334, 335, 338, 340, 344, 345, 348, 349, 356), collected from the same farm, showed 99.46–100% nt and 99.12–100% aa identity with three Romanian strains (ROM-9 to ROM-11). In cluster 4, one Ukrainian strain (UKR-1) showed 98.78% nt and 99.12% aa identity with a Hungarian strain (HUN-296) and one Russian strain (RUS1) showed 86.86% nt and 90.35% aa identity with three Hungarian strains (HUN-142, 143, 144). In some cases, the closest relatives of study strains were found to be ARVs from other continents. A five-week-old unvaccinated broiler flock showed clinical signs characteristic to ARV infection, such as uneven growth, ruffled feathers and diarrhea; moreover, ten percent of males showed signs of lameness. Necropsy revealed greenish swelling accompanied by inflammation of tendon and in some cases rupture of tendon in the hock joint. Interestingly, we were not able to isolate ARV from the tendon samples of these birds, at the same time, reovirus was detected in the intestine samples originating from the same flock. The isolated strains (e.g., HUN-107, HUN-109, HUN-112 and others) showed a close relationship (nt 91.3–93.2%, aa 90.8–91.7%) with strains ISR528, ISR529 and ISR5212 isolated in Israel in 2005 in association with tenosynovitis and with strain USP_BR_1365-2 isolated in Brazil in 2019 in association of runting-stunting syndrome (91.3% nt, 90.8% aa) [5,23]. Moreover, a large number of isolates from healthy flocks (e.g., HUN-168, HUN-178, HUN-182 and others) were also closely related to strains ISR528, ISR529 and ISR5212 and USP_BR_1365-2. The isolates HUN-129 and HUN-131 originating from 15-day-old broilers suffering from malabsorption syndrome clustered with strain 17227-M-10 (nt, 96.5–96.7%, aa, 94.3–94.7%) which was isolated from the same farm in Hungary, but in 2010. The clinical signs of diseased birds in 2010 were necrotic enteritis, pericarditis and nephritis [24]. Similarly, three nearly identical strains (HUN-142, HUN-143 and HUN-144) were isolated from a 42-day-old flock showing no clinical signs and these isolates were closely related to another Hungarian strain, 17203-M-06, isolated from diseased chicken flock in 2006 (nt, 95.3%; aa, 95.2%). In the 2006 cases, airsacculitis, necrotic enteritis and pericarditis were observed after necropsy [24]. 

Vaccination history was available for breeder flocks, where immunization programs started with the live S1133 vaccine at the age between 5 and 9 weeks, and a booster was given usually at the age of 12–14 weeks with inactivated vaccine containing strains 1733 and 2408. Broiler flocks were not vaccinated, they received only maternal antibodies from their vaccinated parents. In a single flock, one cluster 4 and two cluster 1 isolates were detected, the latter composing a common genogroup with vaccine strains, but sharing only 73–74% nt and aa identities with S1133 (as well as with inactivated vaccine strains). Eleven isolates belonging to cluster 2 and cluster 5 were isolated from vaccinated breeders, while 61 isolates belonging to cluster 1, 2, 3 and 4 were isolated from unvaccinated broilers. 

## 4. Discussion

In this paper, the results of a single-year survey of ARV infections are presented from the East-Central region of Europe, predominantly from Hungary. Sampling in our study was part of a routine health survey of chicken flocks; thus, only those flocks were investigated where field visits were performed in this period by collaborating expert veterinarians. The diagnostic workflow was based on virus isolation and RT-PCR detection. Although this protocol may have introduced some bias in the detection success favoring to ARV strains that can be more readily isolated in cell culture, the number of isolates from multiple locations permitted us to describe considerable genetic diversity among co-circulating strains. With this approach we may have missed additional ARV-positive flocks; however, the fact that the survey was not syndrome-specific helped us to understand that ARVs circulate in healthy flocks at a large proportion in Hungary and perhaps in neighboring countries. The circulation of ARVs in healthy flocks may increase the risk for symptomatic infection to occur when breeding or fattening circumstances change. All ARV strains were isolated from the digestive tract (proventriculus, small intestine and caecum) regardless of the observed disease. ARVs from lame birds were isolated also from the gut and not from the tendon or synovial fluid. Although the finding that a large number of gut samples contained infectious ARV is not surprising, the failure of ARV isolation from the tendon of birds with tenosynovitis is somewhat unexpected and this finding opposes to those reported by other groups [7,8,25]. It is possible that in this study the tendon samples were accessed at a later stage of disease when infectious viruses were absent in the synovium, or, other factors (perhaps suboptimal transport or storage conditions) prevented the isolation of ARVs in the laboratory. 

A main objective of the study was the description of strain diversity as recent reports from parts of the world imply that probable vaccine breakthrough events are associated with emerging strains that often share low genetic similarity with vaccine strains [7]. Despite the short study period, considerable countrywide diversity of ARV strains was seen in Hungary indicating that multiple genotypes circulate simultaneously. During the short survey period we identified the five globally common genetic clusters [26,27]. These five clusters were defined as Kant and co-workers suggested [20]. We did not ignore the efforts by other authors who wished to extend this classification scheme with a sixth genotype [2,6,21,22]; however, relevant studies in this respect are controversial and, in some studies, the newly assigned sixth genetic cluster is clearly a divergent branch within one of the originally identified genetic clusters (Figure 4). Thus, in the absence of agreement concerning classification of ARVs, we chose to use the original scheme recommended in the early 2000s and this decision was supported by the finding that all study strains could be classified into the extant clusters reported by Kant et al. [20]. 

Most of the isolates belonged to cluster 2 (*n* = 43); this was followed by cluster 4 strains (*n* = 24), then cluster 1 and 5 strains (with two isolates each) and cluster 3 with a single identified strain. 

Furthermore, when analyzing the strains’ origin within each genotype, close relatedness was seen between some Hungarian cluster 2 and cluster 4 strains and those originated from Romania, Russia and Ukraine, respectively. Poultry trade between these countries may have contributed to the spread of strains with shared σC gene, however, given that reoviruses readily undergo reassortment, a whole genome analysis would be needed to assess the true genetic similarity between strains and potentially imply trade-associated spread of ARV clones. Simple methods to generate reoviral whole genome sequences are available and these procedures can be readily integrated in laboratory workflows whenever the origin and transmission routes of ARVs becomes a matter of scientific interest or legal debate [24,28].

Literature data show that cluster 1 strains are globally common [4,26]. In our study, cluster 1 strains were found in two out of 72 isolates (3%) with available sequence data, furthermore, these two cluster 1 isolates were only distantly related to S1133. Additionally, cluster 1 strains are relatively often reported from wild birds. Of interest, some of these wild bird origin ARV strains are closely related to the vaccine strain, S1133, which is widely used in live attenuated vaccines [29,30]. These findings raise some intriguing questions concerning the risk of infection of wild birds in close contact with flocks of chicken vaccinated with S1133 and their possible role in perpetuating of chicken-associated ARV strains among birds in nature. Nonetheless, vaccine-derived (i.e., S1133-like) reoviruses were not isolated in this study, a finding that indicates that the length of vaccine strain shedding did not overlap with sampling, or, there might have been other factors that prevented the isolation of vaccine strains. Similar to cluster 1 strains, cluster 2 to cluster 5 strains are also geographically widespread, and strains belonging to these four clusters were reported from all five continents [26]. Outbreaks in America have been dominated by cluster 1, 2 and 5 strains. Interestingly, only a few cluster 4 isolates were identified in South America, while in North America cluster 4 strains were detected in high numbers, even if it was not the dominant genotype [2,4,6,20,21,22,23,26,31,32]. From European countries (such as, Hungary, Germany, Greece, Poland, Romania, Russia, Spain, Ukraine and the Netherlands), mostly cluster 4 isolates were reported, and clusters 4 and 2 strains were predominant in Asia and the Middle East [5,11,20,24,26]. 

In this study, ARVs were detected from birds with clinical presentation of runting-stunting syndrome, lameness, uneven growth rate and increased mortality in the flock. We could not find any association between the phylogenetic classification of the isolates and the disease caused by ARV, although the number of samples was relatively low (41 genotyped ARV strains from 10 flocks with ARV associated syndromes). Similar findings were published by Kant et al. (2003) in their milestone genetic characterization study, and a lack of association between pathology and σC based phylogeny was reported in subsequent studies [2,5,20,22,26]. Thus, accumulating evidence suggests that σC genotype specificity may not be at all or may not be the only viral determinant associated with a particular ARV-related disease. It is likely that other genetic features, including some relevant mutations in key genes, or, the constellation of a particular set of ARV gene variants may contribute to the pathogenicity. There is no doubt that ARV research would benefit from the future development of a plasmid-based reverse genetics system and the toolset of site-directed mutagenesis. 

## 5. Conclusions

In conclusion, this study reaffirms the worldwide distribution of the major genetic clusters of chicken origin ARVs. The diversity of strains isolated from unvaccinated broilers was greater than those detected from vaccinated animals (i.e., broiler breeders), but further investigations are needed to understand the relative importance of different vaccination schemes on the selection of novel strains in different chicken breeds.

## Figures and Tables

**Table 1:** Results of virus isolation from broiler samples according to the sample type.

Sample (Organ)	No of Samples		Sum
	CPE−	CPE+	
Intestine	215	103	318
Tendon	57	-	57
Proventriculus	3	3	6
Yolk sac	5	-	5
Kidney	2	-	2
Trachea	2	-	2
Liver	1	-	1
Sum	285	106	391

**Table 2:** Results of virus isolation from different sample types.

Breed	Symptoms	Sample (Organ)	No. of Samples	
			ARV−	ARV+
Layer				
	fibrinous peritonitis	intestine	6	
Broiler				
	healthy	intestine	91	31
		tendon	3	
		proventriculus	1	1
	runting-stunting syndrome	intestine	31	22
		liver	1	
		proventriculus	2	1
	uneven growth rate	intestine	23	15
		tendon	1	
		trachea	2	
		kidney	2	
	diarrhoea	intestine	5	
	mortality	proventriculus		1
		intestine	3	
	lameness	tendon	2	
	n.a.	intestine	10	
Broiler breeder				
	healthy	intestine	44	3
		tendon	18	
		yolk sac	5	
	lameness	intestine	21	3
		tendon	26	
	runting-stunting syndrome	intestine	5	5
		tendon	7	
Sum			309	82

**Table 3:** Average age of ARV-positive flocks in different health status.

Health Status	Mean Age of Flock (Weeks)	
	Broiler	Broiler Breeder
Healthy	4	18
Mortality	4	-
Runting-stunting syndrome	3	5
Lameness	-	5
Uneven growth rate	4	-

## Data Availability

The datasets generated during the current study are available in the GenBank under the accession numbers OP816667–OP816738.

## Supplementary Materials

The following supporting information can be downloaded at: https://www.mdpi.com/article/10.3390/ani13132137/s1.
